# Syphilitic aortic aneurysm with a pulmonary lesion: a case report and literature view

**DOI:** 10.1186/s40064-016-3397-5

**Published:** 2016-10-06

**Authors:** Wei Li, Xiu-Jun Yin, Hua-Ying Liu, Rong Yang

**Affiliations:** 1 Intervention Treatment Center, West China Hospital, Sichuan University, Chengdu, 610041 China; 2 Department of Infectious Disease, West China Hospital, Sichuan University, Chengdu, 610041 China; 3 Department of Thoracic Surgery, West China Hospital, Sichuan University, Chengdu, 610041 China; 4 Department of Neurology, West China Hospital, Sichuan University, No. 37, GuoxueAlley, Chengdu, 610041 China

**Keywords:** Syphilitic aortic aneurysm, Lung cancer, Endovascular stenting, Angiography, Surgery

## Abstract

**Introduction:**

Syphilitic aortic aneurysm (SAA) is caused by tertiary stage of syphilis infection. As the wide application of penicillin, this complication is becoming rarer than before. The SAA with lung cancer is a very rare disease in patient.

**Case description:**

A 55-year-old male was admitted to the hospital complaining “progressive hoarseness for 3 months” and the patient has been diagnosed with syphilis after specific blood exams, computed tomography angiography (CTA) and 3dimensional (3D) reconstructions of cardiac vessels. Chest computed tomography displayed an anomalous soft tissue mass with slightly lobular borders in the peripheral segment of the left lower lobe. According to the aneurysm’s and lung neoplasm’s location, several procedures could be selected such as aneurysm resection with artificial graft replacement or endovascular stenting under angiography. Then, the lesion was removed by lobectomy using video-assisted thoracic surgery.

**Discussion and Evaluation:**

Cardiovascular syphilis remains a major cause of ascending aortic aneurysm. The clinical manifestations of patients with syphilis aortic aneurysm could vary. Aortic imaging is necessary to confirm the diagnosis and to determine the anatomic extent of the aneurysm. The differential diagnosis of the lesion in the pulmonary is mostly the tumor like pulmonary lesion, Pulmonary syphilis. Some studies showed that thoracic aortic aneurysm has been reduced by using penicillin. However, penicillin therapy alone is not always sufficient in recent years. The serologic response to treatment is more significant and faster in patients treated with the enhanced regimen compared to patients treated with the standard penicillin regimen.

**Conclusions:**

Syphilitic aortic aneurysm with lung cancer is a rare disease in patient. Chest CT and CTA scans are able to indicate the presence of SAA. Pathological analysis is an effective method to clarify the diagnosis of the lung lesion. The interventional therapy and surgery are regular treatment method for SAA and pulmonary neoplasm.

## Background

Syphilitic aortic aneurysm (SAA) is caused by tertiary stage of syphilis infection. As the wide application of penicillin, this complication is becoming rarer than before. However, syphilitic aortitis (SA) dose still exist in developed and developing country (Vaideeswar 2010; Roberts et al. 2009). At the beginning, inflammation starts from outermost layer of the blood vessel, including the vasa vasorum that supply the aorta itself with blood. As the situation worsens, the vasa vasorum undergo hyperplastic thickening of their walls thereby restricting blood flow and causing ischemia of the outer two-thirds of the aortic wall. If it continues progressing, syphilitic aortitis leads to an aortic aneurysm. Recognition of cardiovascular syphilis is important because in addition to the broader management of aneurysmal disease, it warrants antibiotic therapy for which penicillin is preferred (Mohamed Sarjun Basha et al. 2016; Beppu et al. 2014). Pulmonary lesions in patients with tertiary syphilis are considered to be of syphilitic origin if other causes can be excluded and particularly if the lesion disappears after antisyphilitic treatment (de Almeida Feitosa et al. 2015). However, the SAA with lung cancer is a very rare disease in patient. Herein, we report a very rare case of SAA with a neoplasm located in the left lower lobe in a 55-year-old man.

## Case report

**Fig. 1:**
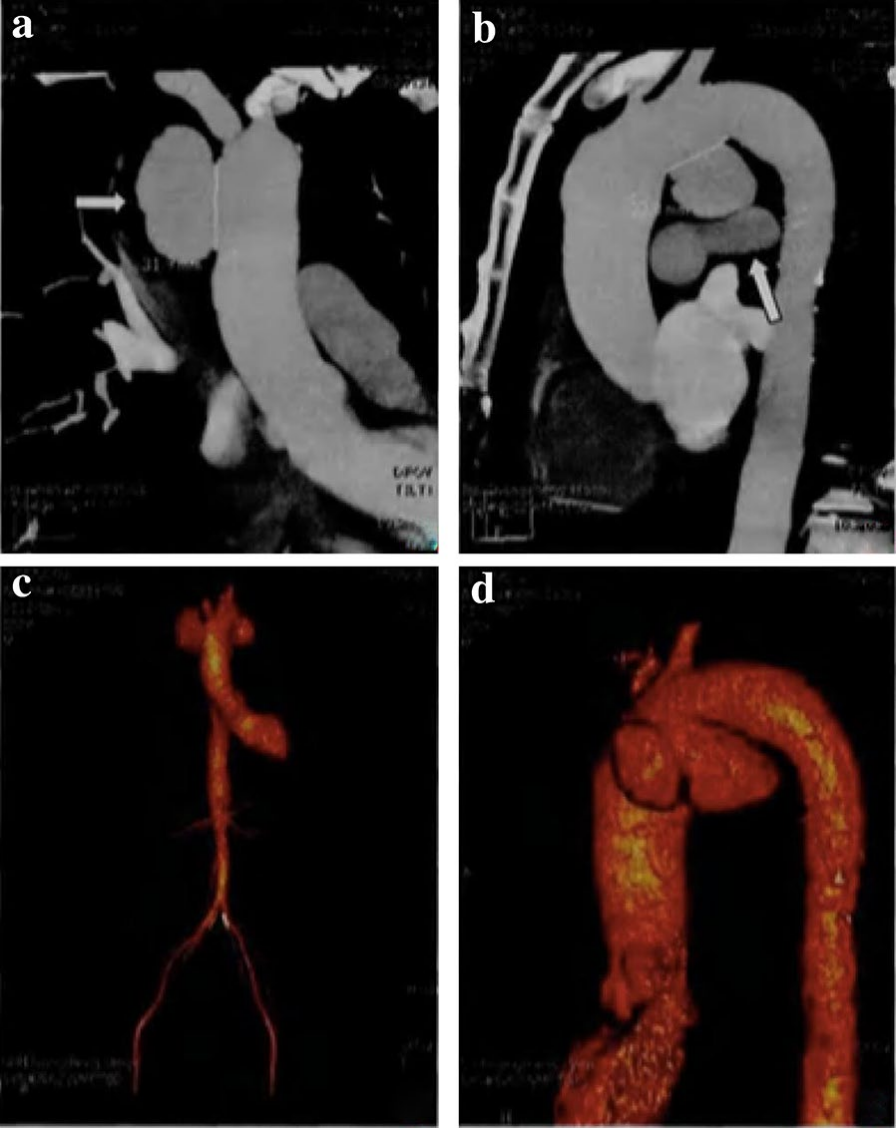
CTA and 3D reconstruction of the whole aorta. **a** and **b** showed multiple tumor-like bulges in the dilated ascending aorta and the arch of aorta with calcification lesions in the aortic wall. The maximum cross-sectional diameters of the dilated segments were 49.1, 33.9 and 19.2 mm. **c** and **d** showed the 3D reconstruction imaging of whole aorta before treatment

**Fig. 2:**
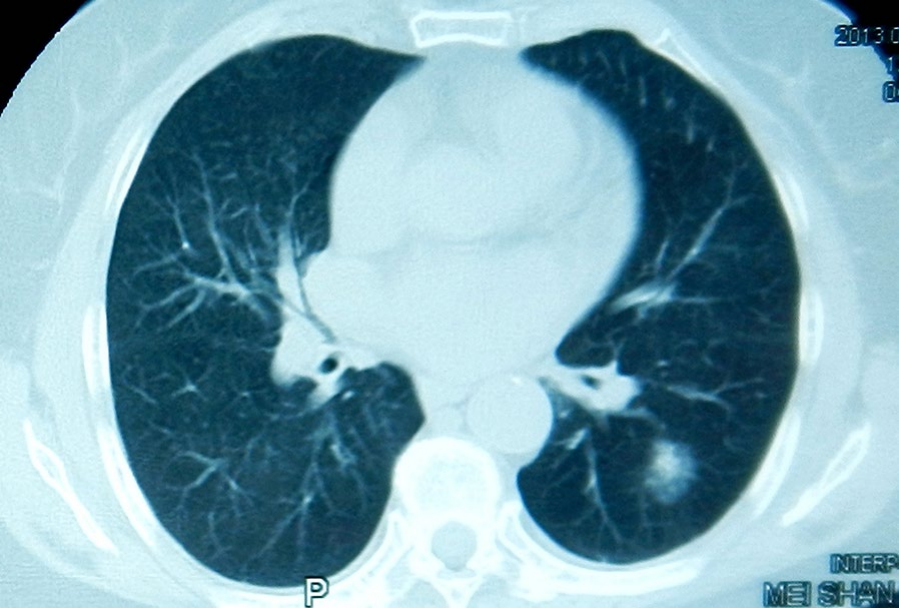
Chest CT of the patient. An anomalous soft tissue mass with slightly lobular borders was located in the segment of the left lower lobe

**Fig. 3:**
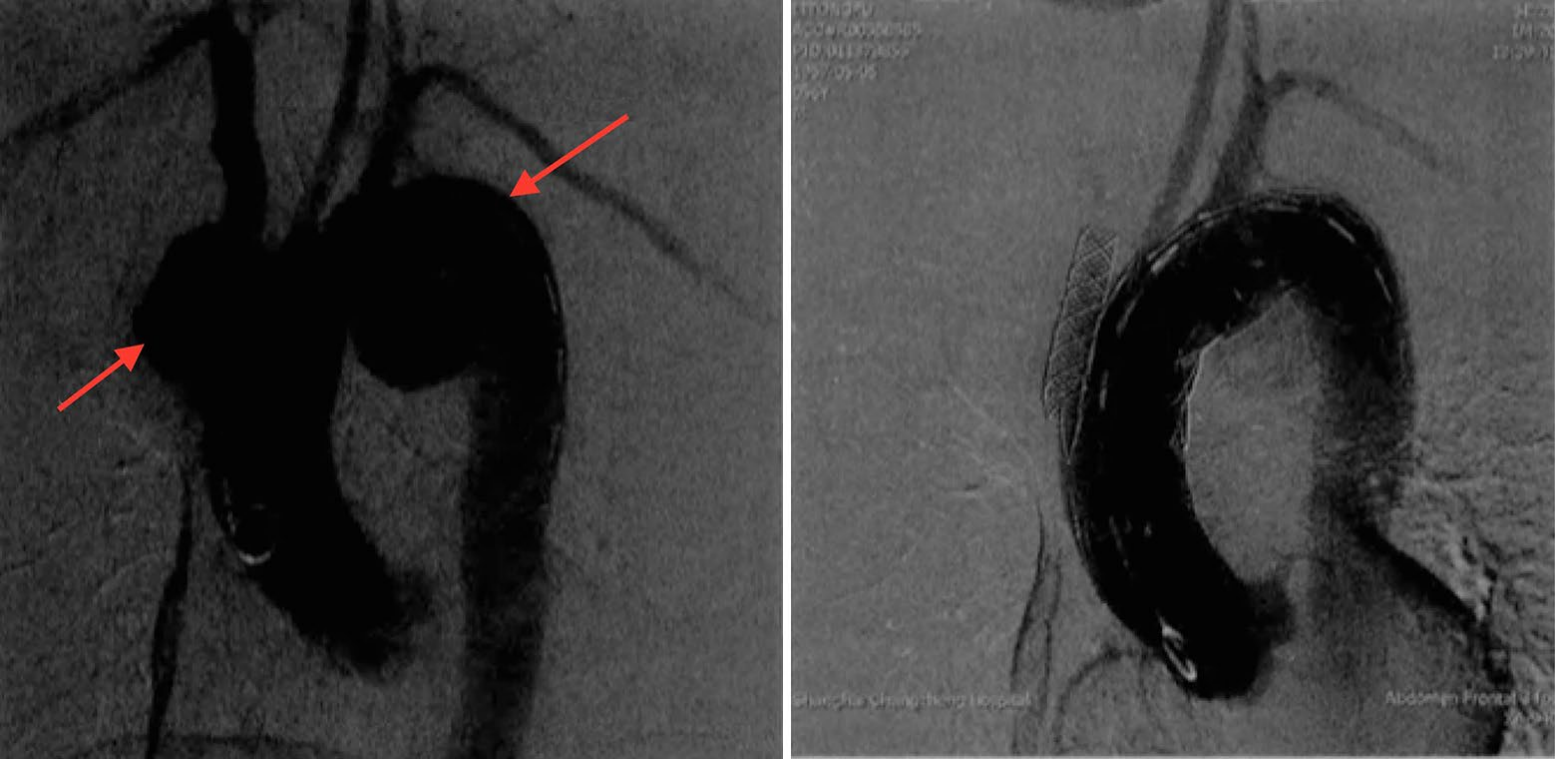
Angiography of the aorta before and after the procedure. Remarkable cystic dilation of ascending aorta and aortic arch is indicated with *arrow*. After the stent implanted, the multiple tumor like bulges of the aorta completely disappeared

A 55-year-old male was admitted to the hospital complaining “progressive hoarseness for 3 months”. He denied symptoms, including chest pain, cough, dry cough, and dyspnea. He was a nonsmoker and had no exposure to any environmental fumes or dust. He had no history of interest or drug allergies. Physical examination revealed normal breathing sounds in both lung fields and heart rate was 80/min and blood pressure was 130/80 mmHg. Auscultation of the heart revealed systolic murmur at aortic area. Laboratory findings were within normal limits. In these examinations, hematology test results and biochemistry test results were within regular levels. Blood tests revealed positive results of treponemalpallidum particle agglutination (TPHA) and fluorescent treponemal antibody absorption test (FTA-Abs) and HIV test was negative. Cardiac ultrasound revealed ectasia of aorta ascendens. So the computed tomography angiography (CTA) and 3dimensional (3D) reconstructions of cardiac vessels were performed (Fig. 1). Chest computed tomography (CT) displayed an anomalous soft tissue mass with slightly lobular borders in the peripheral segment of the left lower lobe (Fig. 2). The mass measured 2.5 cm diametrically. Based on the symptoms, blood results and the CT images, diagnosis is confirmed as syphilitic aortic aneurysm. As the penicillin skin testing was positive, patient was given cefatriaxone intravenously for 14 day (0.2 g on day 1, 0.4 g on day 2, 0.8 g on day 3 and then 2 g per day). The patient was then prepared for the interventional therapy. Angiography of the aorta showed the procedure had been successfully performed (Fig. 3). However, 20 days later, the chest CT still revealed the neoplasm at the same location.

As a diagnosis of the lung lesion was not established through imaging, surgery was scheduled. We approached the tumor, which was removed by lobectomy using video-assisted thoracic surgery (VATS). The surgery was performed in the lateral decubitus position, utilizing a three-port, left-sided VATS approach under general anesthesia administered through a double-lumen endotracheal tube. The tumor located at the left lower lobe had a diameter of about 2.4 cm. At last, the resection of the left lower lobe was made. 1 cm^2^ of the tissue was taken out with a biopsy forceps from the tumor for quick frozen pathology, which was pathologically diagnosed as adenocarcinoma. The postoperative course was ordinary. The patient was discharged 5 days after the operation with no complication. He has been followed up for 8 months without evidence of recurrence.

## Discussion

Cardiovascular syphilis generally manifests about 15–30 years after the initial infection (Beppu et al. 2014). In particular, aortic aneurysm is the most common complication of syphilitic aortitis, and the ascending aorta is the segment most commonly affected (50 %), followed by the arch (35 %) and the descending aorta (15 %) (Sato et al. 2014). In Roberts et al. study (Roberts et al. 2015), they emphasized that syphilis remains a major cause of ascending aortic aneurysm. From January 1, 2009, to December 31, 2014, they studied 23 patients who had resection of an ascending aortic aneurysm that again histologically had classic features of syphilitic aortitis. All 23 patients were found to have syphilitic aortitis grossly and histologically.

The clinical manifestations of patients with syphilis aortic aneurysm could vary. Most intact aortic aneurysms do not produce symptoms. As they enlarge, symptoms such as chest pain and back pain may develop. In patients presenting with aneurysm of the arch of the aorta, a common sign is a hoarse voice from stretching of the left recurrent laryngeal nerve. Rarely, clotted blood which lines most aortic aneurysms can break off and result in an embolus.

Aneurysms can be found on physical examination. Aortic imaging is necessary to confirm the diagnosis and to determine the anatomic extent of the aneurysm. The principal causes of death due to thoracic aneurysmal disease are dissection and rupture. Once rupture occurs, the mortality rate is 50–80 %, and most deaths in patients with the Marfan syndrome are the result of aortic disease (Saleem et al. 2004).

Radiological evidence of a round pulmonary lesion, accompanied by slight fever, loss of weight, and dorsal pains, may lead to a differential diagnosis of tuberculosis, pulmonary infarct, mycotic or bacterial abscess. When patients were diagnosed as aortic aneurysm, clinicians were recommended to perform TPPA, TPHA and FTA-ABS tests both from blood and cerebrospinal fluid (de Souza-Thomas et al. 2005). To establish the diagnosis of aortic aneurysm, CTA is recommended. Although the majority of aortic aneurysm is caused by atherosclerosis, patients with cardiovascular symptoms who were under 50 without history of artery atherosclerosis should remain cautious over syphilitic aortic aneurysm (Lopes et al. 2009). When the diagnosis of syphilis aortic aneurysm is established, penicillin should be given to the patients. To avoid aneurysm rupture, medicine should be given to patients to control blood pressure and anti-platelet aggregation.

The differential diagnosis of the lesion in the pulmonary is mostly the tumor like pulmonary lesion, Pulmonary syphilis. Pulmonary manifestations of acquired syphilis can be of the bronchitic type observed during the secondary stage or tertiary stage and consist either of solitary lesions with the appearance of an abscess or of multiple lesions which are frequently of smaller size and do not produce clinical symptoms (Schibli and Harms 1981). The diagnosis of pulmonary syphilis may be established by the following criteria: First, other causes of chronic lung disease should be excluded by intensive clinical and laboratory study. Second, biopsy material should show the histological changes of a gumma. Since spirochetes can rarely be demonstrated in a syphilitic gumma, other known causes of chronic granulomatous inflammation such as mycotic and mycobacterial agents must be excluded by appropriate histological and cultural studies. Third, the lesion should resolve after antisyphilitic therapy, usually with penicillin (Nunnelee 2007).

Indications for surgery treatment of aortic aneurysm are as followed: (1) If the aneurysm has a rapid growth: superior or equal to 5 mm every 6 months or 10 mm every year; (2) The diameter of ascending aorta or sinuses Valsalva is larger than 55 mm, regardless of symptoms; (3) The diameter of abdominal aorta larger than 50-55 mm; (4) Saccular aneurysm; (5) Symptomatic abdominal aorta aneurysm (Acar et al. 2012; Ben Ahmed et al. 2013; Sekine et al. 2015). Endovascular stenting under angiography must not be realized during the inflammatory phase of the syphilitic aortic aneurysm.

Some studies showed that thoracic aortic aneurysm has been reduced by using penicillin and improvement of public hygiene in developed countries. In recently studies by Zhou et al. and Drago et al., however, they evaluated the efficacy of penicillin in preventing late syphilis complications and found that penicillin therapy alone is not always sufficient to prevent cardiovascular and especially neurological complications (Drago et al. 2012, 2016a, 2016b; Zhou et al. 2012). Evidence that up to 5 % of immunocompetent patients treated with benzathine penicillin G (BPG) experience treatment failurehas wavered confidence in BPG as the gold standard therapy for early and late latent syphilis (Drago et al. 2016c; Ghanem et al. 2006). Trying to prevent long-term complications, Drago et al. adopted in the last 5 years an enhanced therapy for early syphilis that uses doxycycline and ceftriaxone in addition to BPG (Nau et al. 2010). In fact, these treponemicidal antibiotics can penetrate all tissues more effectively than BPG and might be considered as a completion of the standard BPG therapy (Drago et al. 2016a).

The serologic response to treatment reported in such study was more significant and faster in patients treated with the enhanced regimen compared to patients treated with the standard penicillin regimen. Moreover, during the follow up, long-term complications have never been observed and no systemic alterations related to syphilis have been detected at echocardiography or at neurologic examination in patients treated with the enhanced regimen. These data are very encouraging considering that in another study, neurologic and cardiovascular abnormalities have been found in patients with early syphilis over-treated for 2 consecutive years with a total of 60 MU of BPG already after 5 years of followup (Zambon et al. 2010; Dietrich et al. 2014). In fact, 35 % of them revealed neurologic alterations and 19 % cardiovascular complications, confirming that BPG alone may be inadequate, in some patients, in preventing late complications (Drago et al. 2012; Cheng et al. 2014).

In conclusion, Syphilitic aortic aneurysm with lung cancer is a rare disease in patient. Chest CT and CTA scans are able to indicate the presence of SAA. Pathological analysis is an effective method to clarify the diagnosis of the lung lesion. The interventional therapy and surgery are regular treatment method for SAA and pulmonary neoplasm. Pulmonary syphilis is the most common differential diagnosis for the patient with syphilis. To facilitate the preoperative diagnosis and avoid the misdiagnosis of such rare disease, more cases will need to be reported.

## Abbreviations

CT: computed tomography
CTA: computed tomography angiography
FTA-Abs: fluorescent treponemal antibody absorption test
3D: 3dimensional
SAA: syphilitic aortic aneurysm
SA: syphilitic aortitis
TPHA: treponemal pallidum particle agglutination
VATS: video-assisted thoracic surgery
